# Isolated Uvulitis in a Patient After Smoking Fentanyl

**DOI:** 10.7759/cureus.38109

**Published:** 2023-04-25

**Authors:** Cayla Fappiano, Brannon L Inman, Rachel E Bridwell

**Affiliations:** 1 Emergency Medicine, Brooke Army Medical Center, Fort Sam Houston, USA

**Keywords:** drug-related side effects and adverse reactions, acute pharyngitis, fentanyl, airway compromise, uvulitis

## Abstract

Isolated uvulitis is a rare but potentially devastating condition that can result in airway compromise. Etiologies include infection, trauma, allergy, primary angioedema, immunologic disorders, and inhalation injury. Uvulitis has been previously reported as a reaction to inhalation of cannabis, crack cocaine, and mephedrone. We present a case of isolated uvulitis with concerns for impending airway obstruction in a patient after smoking fentanyl. While a sore throat is a common chief complaint among ED patients, emergency providers should consider uvulitis within this deadly differential.

## Introduction

Isolated uvulitis is an infrequently reported condition that can rapidly lead to airway compromise if untreated [1,2]. While the infection is a common cause, other etiologies include trauma, allergy, primary angioedema (Quincke’s disease), immunologic disorders, and inhalation injury [2-15]. The inhalation of cannabis, crack cocaine, and mephedrone has previously been described as an etiology of uvulitis [11-15]. We present a unique case of isolated uvulitis in a patient after smoking fentanyl.

## Case presentation

**Figure 1:**
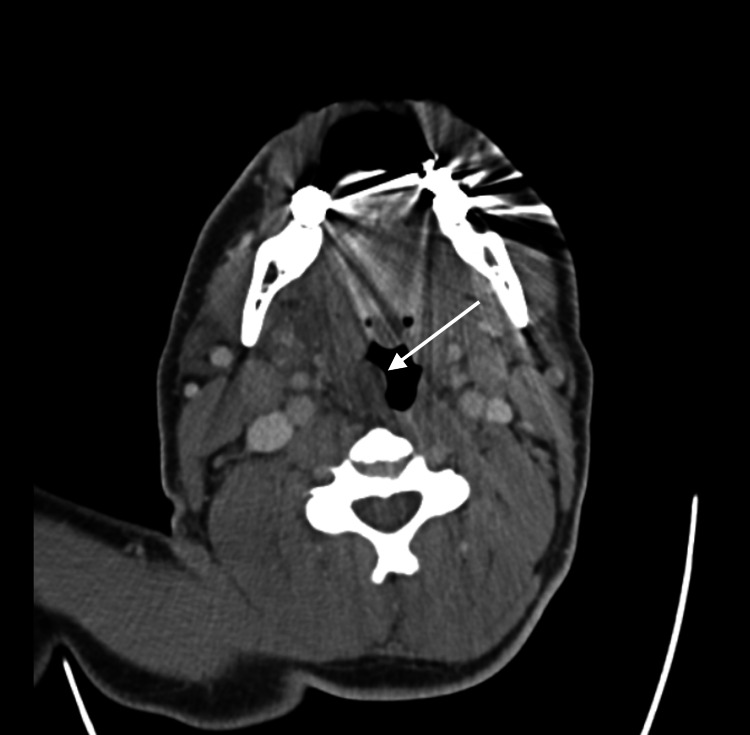
Axial CT of the neck with intravenous contrast demonstrating isolated uvulitis without tonsillitis or other evidence of pharyngitis

A 42-year-old male with known fentanyl and methamphetamine use presented to the ED complaining of sore throat, odynophagia, dysphonia, and voice changes after smoking fentanyl 12-14 hours prior. Due to worsening odynophagia, the patient developed difficulty tolerating oral secretions. He smoked cigarettes and endorsed a history of intravenous methamphetamine use but denied other recent drug use by any route within the past week. A review of systems was otherwise negative with no trismus or nuchal rigidity. Initial vital signs included a blood pressure of 146/72 mmHg, heart rate of 74 beats per minute, respiratory rate of 18 breaths per minute, oxygen saturation of 96% on room air, and an oral temperature of 99.0 degrees Fahrenheit. Physical exam was notable for an enlarged, erythematous uvula with clear extension past the tongue base without deviation. The patient was noted to have poor dentition and no intraoral tenderness nor submandibular brawny induration. Laboratory evaluation demonstrated a white blood cell count of 11.7 x 10^9/L and a urine drug screen positive only for opiates. Hemoglobin, platelets, and basic metabolic panel were otherwise within normal limits. CT of the neck with intravenous contrast demonstrated isolated uvulitis without tonsillitis or other evidence of pharyngitis though bilateral reactive cervical lymph nodes were noted (Figure 1). The patient received 10 mg of dexamethasone and 3 g of ampicillin-sulbactam intravenously. The patient was transferred to a tertiary care center for otolaryngology consultation and close airway observation.

## Discussion

Isolated uvulitis is an infrequently reported condition that can lead to airway occlusion if left untreated [1-2]. While a sore throat is the most common chief complaint associated with uvulitis, patients may also experience odynophagia, dysphagia, and dyspnea [1]. Uvular inflammation is more commonly associated with other inflammatory oropharyngeal diseases like pharyngitis [2]. Infectious causes are most commonly Haemophilus influenzae type B and group A *Streptococcus* spp., occurring predominantly in children. Uvulitis is more commonly non-infectious in adults, with etiologies including trauma, allergy, primary angioedema, immunologic disorders, inhalation injury, and drug use [2-15].

Uvulitis secondary to drug use has previously been described after cannabis, crack cocaine, and mephedrone inhalation [11-15]. Mephedrone was nasally inhaled in powder form, while cannabis and crack cocaine were smoked [11-15]. Some drug-related cases have been postulated to be type-1-hypersensitivity reactions with pale, translucent-appearing uvulas [11-13]. The above patient presented with an enlarged, erythematous uvula, not consistent with angioedema. Direct thermal injury is thought to be the cause of uvular edema in the setting of some drug use; marijuana has been postulated to cause a thermal injury because it burns at a higher temperature than tobacco [14-15]. However, the smoke temperature depends not only on the flash point of the substance but also on the other heating conditions of the substance including the method of smoking (rolled cigarette, pipe, vape), the use of a filter, and the other contents of a cigarette [16]. Fentanyl itself has not previously been reported as a potential cause of isolated uvulitis.

Addressing uvulitis hinges on airway management; significant edema may necessitate the use of a flexible intubating endoscope or nasotracheal intubation [2]. Immediate administration of antibiotics covering oral anaerobes is a mainstay of treatment, regardless of the presence of systemic signs like fever, as delaying therapy increases the risk of airway compromise [2,3]. Evaluating for concurrent epiglottitis on lateral neck films or deep space neck infection on a CT scan may be warranted if the patient can tolerate supine positioning without airway difficulty [4]. Suspected allergic uvulitis should be treated with an antihistamine, epinephrine, and steroids [5]. All patients should be observed for airway monitoring with an otolaryngologist consult for severe cases [2].

## Conclusions

Isolated uvulitis is an uncommon but potentially life-threatening cause of sore throat with many potential etiologies that can lead to airway compromise. Prompt identification and treatment of uvulitis are imperative in the ED. Considering inhalation injury due to substance abuse is an important element of the patient’s history that may help guide treatment. Uvulitis should be included in the deadly pharyngitis differential for emergency clinicians as a high-acuity, low-incidence disease.

## References

[1] McNamara RM (1994). Clinical characteristics of acute uvulitis. Am J Emerg Med.

[2] Hawke M, Kwok P (1987). Acute inflammatory edema of the uvula (uvulitis) as a cause of respiratory distress: a case report. J Otolaryngol.

[3] Brook I (1997). Uvulitis caused by anaerobic bacteria. Pediatr Emerg Care.

[4] Shomali W, Holman K (2016). Concurrent uvulitis and epiglottitis. Cleve Clin J Med.

[5] Nguyen L, Stead TS, Lopez Ortiz C, Gillespie R, Ganti L (2021). Anaphylaxis presenting as uvulitis. Cureus.

[6] Shiber JR, Fontane E (2014). Quincke's disease: isolated uvulitis. West J Emerg Med.

[7] Gilmore T, Mirin M (2012). Traumatic uvulitis from a suction catheter. J Emerg Med.

[8] Peghini PL, Salcedo JA, Al-Kawas FH (2001). Traumatic uvulitis: a rare complication of upper GI endoscopy. Gastrointest Endosc.

[9] Miller RJ, Gerhardt MA (2006). Uvular edema secondary to snoring under deep sedation. Anesth Prog.

[10] Kazi A, Gauthier M, Lebel MH, Farrell CA, Lacroix J (1992). Uvulitis and supraglottitis: Early manifestations of Kawasaki disease. J Pediatr.

[11] Murphy A, Haughey R (2014). Mephedrone-induced uvulitis. Anaesthesia.

[12] Macfarlane R, Hart J, Henry JA (2002). A man with a massive uvula. Lancet.

[13] Welling A (2008). Enlarged uvula (Quincke's oedema)--a side effect of inhaled cocaine?--a case study and review of the literature. Int Emerg Nurs.

[14] Boyce SH, Quigley MA (2002). Uvulitis and partial upper airway obstruction following cannabis inhalation. Emerg Med (Fremantle).

[15] Mallat A, Roberson J, Brock-Utne JG (1996). Preoperative marijuana inhalation--an airway concern. Can J Anaesth.

[16] Mallock N, Pieper E, Hutzler C, Henkler-Stephani F, Luch A (2019). Heated tobacco products: a review of current knowledge and initial assessments. Front Public Health.

